# Knowledge of Regulation of Photosynthesis in Outdoor Microalgae Cultures Is Essential for the Optimization of Biomass Productivity

**DOI:** 10.3389/fpls.2022.846496

**Published:** 2022-04-04

**Authors:** Giorgio Perin, Francesca Gambaro, Tomas Morosinotto

**Affiliations:** Department of Biology, University of Padova, Padua, Italy

**Keywords:** microalgae, photosynthesis regulation, acclimation, photobioreactors, cultivation outdoors

## Abstract

Microalgae represent a sustainable source of biomass that can be exploited for pharmaceutical, nutraceutical, cosmetic applications, as well as for food, feed, chemicals, and energy. To make microalgae applications economically competitive and maximize their positive environmental impact, it is however necessary to optimize productivity when cultivated at a large scale. Independently from the final product, this objective requires the optimization of biomass productivity and thus of microalgae ability to exploit light for CO_2_ fixation. Light is a highly variable environmental parameter, continuously changing depending on seasons, time of the day, and weather conditions. In microalgae large scale cultures, cell self-shading causes inhomogeneity in light distribution and, because of mixing, cells move between different parts of the culture, experiencing abrupt changes in light exposure. Microalgae evolved multiple regulatory mechanisms to deal with dynamic light conditions that, however, are not adapted to respond to the complex mixture of natural and artificial fluctuations found in large-scale cultures, which can thus drive to oversaturation of the photosynthetic machinery, leading to consequent oxidative stress. In this work, the present knowledge on the regulation of photosynthesis and its implications for the maximization of microalgae biomass productivity are discussed. Fast mechanisms of regulations, such as Non-Photochemical-Quenching and cyclic electron flow, are seminal to respond to sudden fluctuations of light intensity. However, they are less effective especially in the 1–100 s time range, where light fluctuations were shown to have the strongest negative impact on biomass productivity. On the longer term, microalgae modulate the composition and activity of the photosynthetic apparatus to environmental conditions, an acclimation response activated also in cultures outdoors. While regulation of photosynthesis has been investigated mainly in controlled lab-scale conditions so far, these mechanisms are highly impactful also in cultures outdoors, suggesting that the integration of detailed knowledge from microalgae large-scale cultivation is essential to drive more effective efforts to optimize biomass productivity.

## Graphical Abstract

How does microalgae photosynthesis respond to outdoor cultivation at industrial-scale?

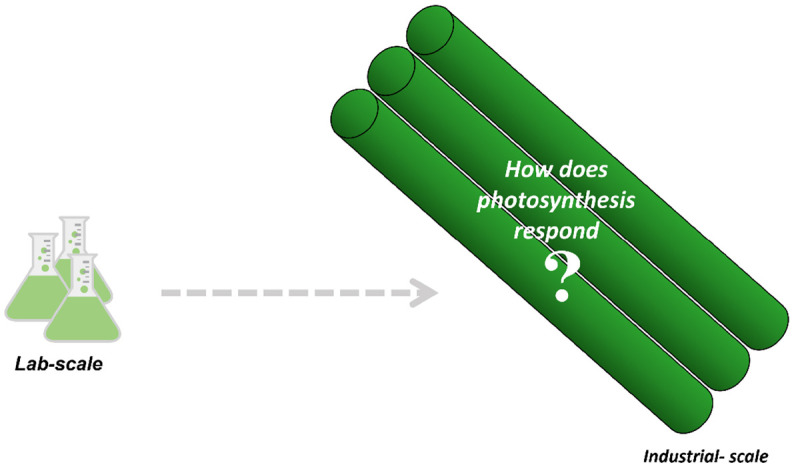

## Introduction

Global demand for products derived from biomass such as food, feed, or fuels is continuously expanding and will continue to grow with the increase of population in the next decades. At the same time, agricultural practices generate several negative impacts on the environment, such as CO_2_ emissions and reduction of biodiversity (Lynch et al., 2021). A major challenge for the future well-being of our society is developing new strategies to improve the current biomass production, while also increasing sustainability and mitigating the emission of greenhouse gases in the atmosphere (Walsh et al., 2015; Huang et al., 2021). One promising possibility is to complement plants cultivation with new biomass sources such as microalgae, diverse, unicellular, eukaryotic photosynthetic organisms that use sunlight to produce biomass and oxygen from carbon dioxide (CO_2_), water, and nutrients. Microalgae can be cultivated in marginal or saline water on unproductive land and thus do not compete with agriculture for arable soil and freshwater, making them a promising resource for highly sustainable production of biomass and various biomaterials (Perin and Morosinotto, 2019b; Fabris et al., 2020).

While many promising microalgae applications have been proposed, the potential of these organisms remains largely underexploited. Significant improvement in strains performances and cultivation technologies is still seminal to reduce biomass production costs and increase competitivity (Perin and Morosinotto, 2019a). Photon-to-biomass conversion efficiency is one of the major parameters impacting microalgae productivity and thus its optimization is strategic for the expansion of microalgae-biomass-based technologies.

Microalgae cultivation is performed in industrial platforms (e.g., photobioreactors, PBRs) with high energy and monetary costs for both building and maintenance. Maintaining a high photon-to-biomass conversion efficiency at an industrial scale is thus essential to guarantee high areal productivity, improving the ratio between production and building/operational costs (Simionato et al., 2013).

**FIGURE 1:**
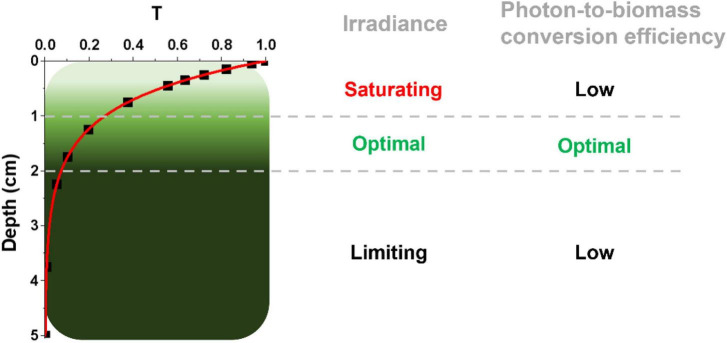
Light distribution in a dense microalgal culture. The light attenuation profile is here expressed as transmittance (T), namely the ratio between transmitted and incident light, and it is described as a function of the depth of the photobioreactor (in centimeters, cm), using experimental data from a *Nannochloropsis gaditana* culture at 1 g L^–1^ concentration in a PBR with a 5-cm depth (Perin et al., 2017b). According to this light attenuation profile, three different regions within the PBR can be identified, distinguished for their irradiance and photon-to-biomass conversion efficiency profiles, as described on the right. It is worth noting that the culture layers more distal from the light source (from the top in this case) have a potential high photon-to-biomass conversion efficiency, which is however not achieved because of the limiting light they experience.

Microalgae at an industrial scale are normally cultivated at high cell densities to maximize biomass productivity, leading to an inhomogeneous light distribution because of cells’ self-shading. As shown in the example in Figure 1, which reports estimations for a culture with a 5 cm depth and 1 g L^–1^ biomass concentration, the first cm of the culture absorbs approx. 60% of incident radiation, whilst most of the remaining 40% of incident radiation is harvested by the second cm of culture, leaving the rest almost in the dark (Perin et al., 2019). The first layer of cells, besides absorbing most of the available radiation, often experiences saturation of photosynthesis and thus exploits harvested light with lower efficiency. An intermediate layer of cells receives optimal light to support growth, leaving however most of the culture volume in light limitation, where energy necessary for cell maintenance can be even larger than the light absorbed, curbing photon-to-biomass conversion efficiency. This phenomenon is particularly impactful in microalgae large scale cultivation, where in most cases optical paths are larger than the example in Figure 1, reaching even 20 cm and more in the case of raceway open ponds (Kim et al., 2018).

Microalgal cultures are actively mixed to improve cells exposition to incident light and to ensure the supply of nutrients and CO_2_ that can be limited by the low diffusion rate in liquid medium. Because of the combination of mixing and shading, cells move between different layers of the mass culture and thus from regions where light is saturating to others where it is limiting and *vice-versa.* Because of these movements, cells are thus exposed to abrupt changes in irradiance, with a millisecond to minutes timescale, depending on the design of the platform of cultivation, e.g., photobioreactors or ponds, respectively (Carvalho et al., 2011).

The impacts of cells shading can be reduced by decreasing the optical path of the cultures, modifying the design of the cultivation platform, using, e.g., thin-layer photobioreactos (Masojídek et al., 2010), and bringing to a reduction in the culture volume per area of cultivation platform. This alone indeed can provide an increase in volumetric yield (Chuka-ogwude et al., 2021), but it also leads to higher building and operational costs that needs to be compensated for by higher productivity (Pruvost et al., 2017). In thin-layer systems, however, the smaller optical path corresponds to a higher biomass concentration, thus still generating a significant shading.

Independently from the specific cultivation systems, in order to improve biomass productivity of microalgae industrial cultivation and make it economically and energetically sustainable, it is thus essential to optimize the light-energy-to-biomass-conversion efficiency in an optically dense culture. One possibility to reach this objective is tuning their photosynthetic properties to cultivation in intensive conditions, where the wild-type (WT) strains isolated in nature may not be optimal to reach maximal productivity (Formighieri et al., 2012). Strains improvement or domestication by the selection of specific genetic features is expected to be seminal for the industrial exploitation of microalgae biomass (de Jaeger et al., 2014; Perin et al., 2015; Perin and Jones, 2019). Genetic modifications of microalgae photosynthetic properties were indeed shown to be beneficial for productivity at the lab scale (Kirst et al., 2012; Perin et al., 2015; Friedland et al., 2019; Negi et al., 2020), while attempts to validate the improved productivity at larger-scale cultivation were successful in some cases but not others (Cazzaniga et al., 2014; De Mooij et al., 2014). The variability of outcomes largely depends on the environmental conditions experienced in large-scale cultivation, which are significantly different from the lab scale and have a major impact on performances of genetically improved strains (Perin et al., 2017a,b). When microalgae are cultivated in PBRs/ponds outdoors, in fact, they are exposed to a complex environment, where the specific features described in Figure 1 are combined with the impact of natural fluctuations of light and temperature, impairing the possibility of extrapolating the impact of genetic modifications at the industrial-scale from simple lab-scale evaluations. A deeper understanding of the complexity of the growth environment in outdoor industrial-scale systems and its impact on microalgae productivity is thus essential to close the current gap between lab- and PBRs-scale growth performances and to translate more effectively the potential of genetic improvement to a more relevant scale of cultivation for microalgae industrial applications.

### The Impact of Short-Term Mechanisms for the Regulation of Photosynthesis on Microalgae Productivity

Photosynthetic organisms are exposed to a highly dynamic environment in nature and evolved multiple regulatory mechanisms of photosynthesis with different activation timescales, enabling responses to short- and long-term light dynamics (Roach and Krieger-Liszkay, 2019). The short-term mechanisms enable a quick response to changes in irradiance and they depend on either macromolecule already present in the cell or pools of molecules rapidly synthesized/degraded upon changes in irradiance. One of the fast responses to excess light is Non-Photochemical Quenching (NPQ), which drives the dissipation of a fraction of the absorbed energy as heat (Ruban, 2016), a mechanism that in microalgae mostly depends on the activity of antenna proteins belonging to the LHCSR/LHCX family (Roach et al., 2020; Blommaert et al., 2021). Another important mechanism triggered by excess light is the synthesis of the xanthophyll zeaxanthin from violaxanthin by Violaxanthin De-Epoxidase (VDE). Zeaxanthin enhances NPQ while also playing a direct role in scavenging reactive oxygen species (ROS) (Dall’Osto et al., 2012), generated from the interaction of excited chlorophyll states with molecular oxygen. When irradiance decreases, zeaxanthin is converted back to violaxanthin by zeaxanthin epoxidase (ZE), increasing light-harvesting ability. Whilst this cycle has been described in diverse microalgae species, from the green lineage (e.g., *Chlamydomonas reinhardtii*) to Eustigmatophytes (e.g., *Nannochloropsis* spp.), the analogous diadinoxanthin-diatoxanthin cycle is instead active in diatoms (Lacour et al., 2020).

The mechanisms described above are mainly active in protecting PSII, yet also PSI can experience radiation damage, when light fluctuations drive to over-reduction (Bellan et al., 2020). Two main mechanisms have been identified to be involved in protecting PSI. The first is called photosynthetic control and it is activated by the proton gradient across the thylakoids membrane generated in excess light. This regulation consists in the inhibition of the activity of the Cytochrome b_6_f that consequently reduces the flux of electrons reaching PSI and thus the risk of over-reduction (Barbato et al., 2020). A second group of mechanisms, cyclic (CEF) and pseudo-cyclic (PCEF) electron flows, avoid PSI over-reduction by activating alternative pathways that recycle electrons back to the plastoquinone pool or Oxygen (Shikanai, 2014). Among the proteins that have been so far identified involved in CEF there are PGR5/PGRL1 (Munekage et al., 2002; DalCorso et al., 2008) and the chloroplast NADH-dehydrogenase-like (NDH-1) complex (Shikanai et al., 1998), whilst for PCEF two alternative pathways have been described so far, namely the Mehler reaction and Flavodiiron proteins (FDP or FLVs) (Shikanai and Yamamoto, 2017; Alboresi et al., 2019).

The impact of short-term mechanisms for the regulation of photosynthesis on biomass productivity in the outdoors environment has been demonstrated in plants, where NPQ, xanthophyll cycle and CEF (Hertle et al., 2013; Nawrocki et al., 2019) were shown to impact fitness (Kulheim et al., 2002). Also genetic optimization of the NPQ response was shown to enable significant improvement in biomass yield in tobacco plants cultivated in the field (Kromdijk et al., 2016). It can thus be expected that photosynthesis regulation is impacting productivity of microalgae cultivated in PBRs/ponds outdoors as well.

**FIGURE 2:**
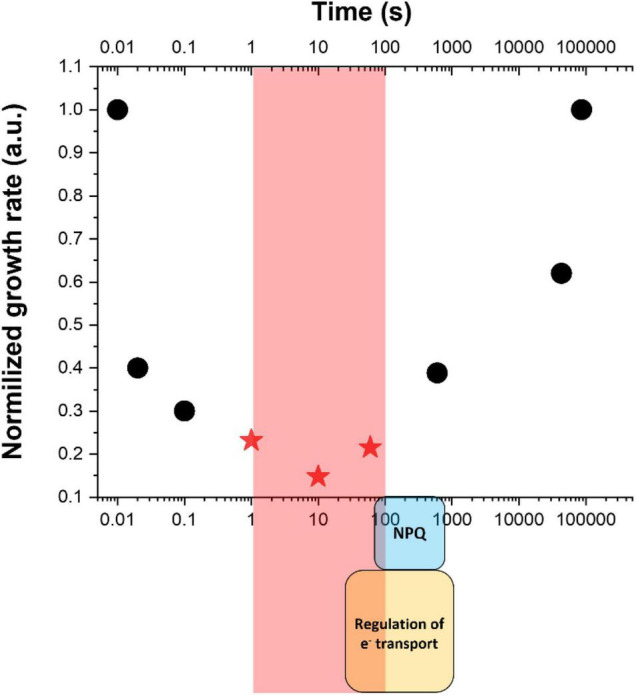
Impact of light fluctuations on microalgae growth. Data indicate the growth rate of *Nannochloropsis gaditana* as a function of light fluctuations with different frequencies, taking that the same optimal amount of photons has been provided to the different cultures [150 μmoles of photons m^–2^ s^–1^ (Sforza et al., 2012)]. Red stars indicate a drop ≈ 85% of biomass productivity and correspond to frequencies in the 1–100 s range (highlighted by a red square) where short-term mechanisms for the regulation of photosynthesis [i.e., regulation of electron (e^–^) transport and NPQ, indicted by a yellow and light blue square at the bottom] are not fully activated. Data are re-elaborated from Sforza et al. (2012) and Bellan et al. (2020).

In the example reported in Figure 2, *Nannochloropsis gaditana* cultures were exposed to dynamic light changes with a highly different frequency of fluctuations, with the saturating light treatment lasting between 0.01 s and hours, while the limiting light being correspondingly six times longer to ensure that the total amount of photons received remained the same (Sforza et al., 2012; Bellan et al., 2020). The integrated photon flux corresponded to optimal light intensity for the species [i.e., 150 μmoles of photons m^–2^ s^–1^ (Sforza et al., 2012)] and nutrients and CO_2_ were supplied in excess. These conditions thus enabled to assess the impact of illumination dynamics resembling those experienced in outdoor PBRs/ponds on microalgae photosynthetic productivity while maintaining all other parameters constant. Results showed that the frequency of light fluctuations alone has a major impact indeed, resulting in over a four-fold decrease in the growth rate and a reduction in the biomass produced by ≈ 85% (Figure 2). This is a clear exemplification that the impact of light dynamics must be strongly considered in all optimization efforts of outdoor productivity.

Results reported in Figure 2 are consistent with similar experiments reported for other species (e.g., *Chlamydomonas reinhardtii*, *Spirulina platensis*, *Chlorella* spp.) where a broad range of light fluctuations was shown to have a strong impact on growth (Phillips and Myers, 1954; Nedbal et al., 1996; Vejrazka et al., 2011; Xue et al., 2011), clearly suggesting that this effect is largely similar in different photosynthetic microorganisms, from cyanobacteria to eukaryotic microalgae species. This observation also suggests that the phenomenon is intrinsic to the photosynthetic machinery and largely dependent on conserved functional mechanisms. Overall, literature data consistently show that very short light flashes do not cause significant damage and that the photons provided can be exploited with good efficiency even if illumination is very intense. This can be explained because intense light flashes are efficiently absorbed by pigments and can initiate photochemical reactions, but light is then switched off before electron flux becomes limited by diffusion-dependent transporters such as that mediated by the plastoquinone pool (Srivastava et al., 1995; Kirchhoff, 2014).

A second common observation is that cells can withstand even a very strong illumination, lasting for several minutes or hours and maintain a good growth rate. When exposed to intense light, regulatory mechanisms are activated, enabling the modulation of the photosynthetic efficiency and the protection from damage by dissipating light excesses as heat.

The most challenging conditions are thus those in which strong light exposure lasts long enough to cause damage but not long enough for the full activation of protection mechanisms. The fastest mechanism known is probably PCET, dependent on FDP whose activation is particularly fast and can provide significant protection in a few seconds after light is switched on (Gerotto et al., 2016). This mechanism, however, is not found in all eukaryotic microalgae, including all secondary endosymbionts like *Nannochloropsis* and diatoms (Bellan et al., 2020). NPQ, on the other hand, requires at least 60 s of intense light to be induced (Bellan et al., 2020). Xanthophyll cycle is instead slower and requires 1–3 min to be activated. Similar kinetics are also needed for the activation of the regulation of CET (Strand et al., 2015). Considering that microalgae are highly diverse and that this variability is also true for regulatory mechanisms of photosynthesis, different species may have slightly different kinetics. Anyhow, present knowledge is quite clear in suggesting that regulatory mechanisms of photosynthesis are not fully activated in the 1–60 s range, where still strong light lasts enough to saturate the electron transport chain and drive damage (Figure 2).

### Long-Term Mechanisms for the Regulation of Photosynthesis in Microalgae

While the mechanisms discussed above are active in the seconds/minutes timescale, the prolonged exposition of cells to a specific irradiance induces the activation of long-term mechanisms for the regulation of the photosynthetic apparatus, such as acclimation, to maintain photosynthesis homeostasis. This phenomenon modulates the composition of the photosynthetic apparatus in response to irradiance intensity and includes the regulation of the content of proteins and other macromolecules (e.g., pigments) (Falkowski and Owens, 1980; Falkowski and LaRoche, 1991; Zou and Richmond, 2000; Walters, 2005). In most photosynthetic organisms, this long-term acclimation mechanism includes a reduction in the chlorophyll (Chl) content and an increase in photoprotective carotenoids (Car) and xanthophylls when exposed to saturating irradiance (Rodríguez et al., 2005). Another common feature of acclimation is the modulation of the photosynthetic capacity with, for example, cells acclimated to high light showing an increased photosynthetic electron transport, as observed in the diatom *Phaeodactylum tricornutum* and in the green microalga *Chlamydomonas reinhardtii* (Nymark et al., 2009; Bonente et al., 2012). This also correlates with an increase of sinks for photosynthetic electrons, such as the Calvin-Benson cycle enzymes, with the enzyme RuBisCO being the main target of the regulation (Fisher et al., 1989; Simionato et al., 2011).

The extent of this acclimation response is exemplified by merging data from *Nannochloropsis gaditana* cultivated at the lab scale in highly different conditions (Supplementary Table 1). These cultures differed for supports of cultivation (flasks, lab-scale photobioreactors with different designs), illumination intensities (10, 100, 400, 1000, 1200 μmol photons m^–2^ s^–1^), media (seawater media F/2, F/2 with additional/depletion of Nitrogen, Phosphorous), presence/absence of external CO_2_ supply, cultivation strategies (batch or semi-continuous), as described in Supplementary Table 1. The comparative analysis of these data, obtained for the same microalgal strain and using a consistent set of analytical methods, highlights the response of microalgae to different growth conditions while minimizing effects due to other parameters, like species/strain and specific cultivation protocols. All data compared are obtained from cells under active growth and do not include cells under prolonged stresses.

**FIGURE 3:**
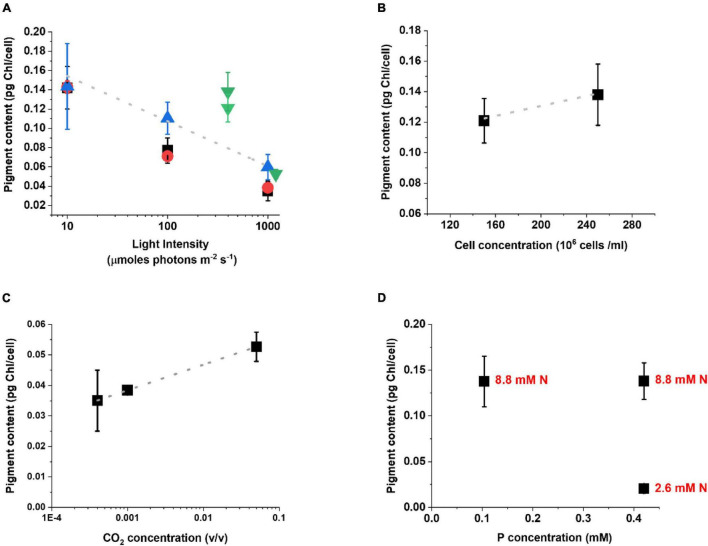
Acclimation response of *Nannochloropsis gaditana* in a plethora of lab-scale growth conditions. Acclimation is expressed as the change in the Chl content [picograms (pg) of Chlorophyll per cell] as a function of different cultivation parameters, as follows: light intensity **(A)**; cell concentration **(B)**; CO_2_ concentration **(C)** and nitrogen (N) and phosphorus (P) availability **(D)**. In panel **(A)** the trend of Chl reduction as the light intensity increases is maintained regardless of the differences in cultivation support, nutrient, and CO_2_ supply. Black squares, data from flasks, both nutrients and carbon are limiting; Red circles, data from lab-scale glass tubes with air bubbling; Blue upward tringles, data from lab-scale glass tubes with air bubbling and excess nutrients; Green downward tringles, data from lab-scale photobioreactors with excess carbon (see Supplementary Table 1 for a complete description of the cultivation conditions). In panel **(D)** black dots indicate the Chl content as a function of P availability, whilst the corresponding concentration of N for each dot is indicated in red. The growth conditions in which these data were collected are summarized in Supplementary Table 1.

The analysis of the pigment content highlights the activation of the acclimation response with a modulation of the Chl content of the cells (Figure 3). Under lower illumination, Chl content increases to maximize light-harvesting efficiency while, when light is intense, Chl content decreases to reduce absorption and avoid eventual photodamage. While this is a common behavior among different photosynthetic organisms (Li et al., 2009; Lepetit et al., 2012; Meneghesso et al., 2016), it is interesting to observe that this correlation emerges despite the differences in other cultivation parameters. Chl content in cells exposed to extremes illumination (10 or 1000 μmol photons m^–2^ s^–1^) is also more similar irrespective of other factors. This probably suggests the existence of a lower and higher limit for Chl accumulation per cell (Figure 3A), whose value is likely species-specific.

While the Chl content is largely influenced by the light intensity (Figure 3A), also other parameters like culture concentration, CO_2_ and nutrient availability show an impact on acclimation, when their influence is specifically analyzed (Figure 3). Cells concentration plays a major effect indeed, since self-shading reduces the average light available to the culture, even if the incident light is equivalent (Figure 3B). A higher CO_2_ availability, on the other hand, stimulates Chl accumulation (Figure 3C), likely because when the Calvin-Benson cycle is more active it re-generates acceptors for photosynthetic electron transport faster, thus enabling the use of photosynthetic excitation energy more efficiently. When the impact of nutrients is considered, Chl biosynthesis is highly dependent on Nitrogen availability and indeed cells with higher supply accumulate more pigments, while they accumulate far less in limiting conditions (Figure 3D). Phosphorous availability, on the other hand, has a smaller influence. In *Nannocholopsis* cultures Phoshoporus deficiency has no major impact on Chl content (Fattore et al., 2021), while significant effects were instead observed in cyanobacteria and other microalgae species (Kokabi et al., 2019, 2020; Solovchenko et al., 2020).

Overall, we observed that different parameters have a large impact on the acclimation of the photosynthetic apparatus (Alboresi et al., 2016; Meneghesso et al., 2016; Perin et al., 2017b; Fattore et al., 2021), highlighting the complexity of this phenomenon.

**FIGURE 4:**
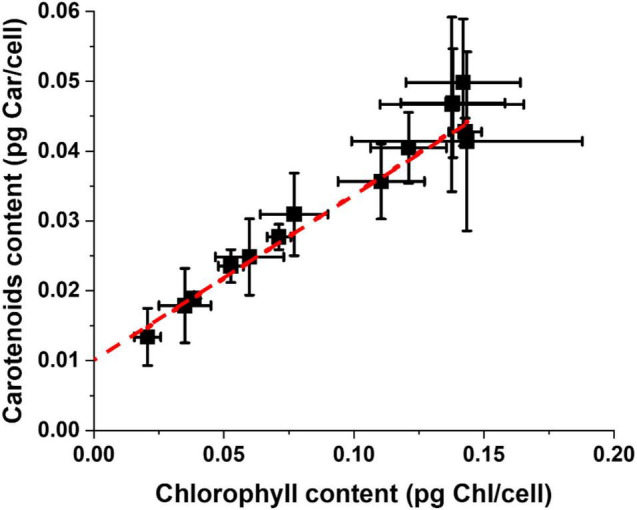
Carotenoids and Chlorophyll content of *Nannochloropsis* cells cultivated in a plethora of lab-scale conditions. Data were treated independently from the environmental parameters of the different growth conditions of Supplementary Table 1.

Carotenoids (Car) are the other major pigments in photosynthetic organisms, active in light-harvesting but also in protection from light excess and their content is also modulated during the acclimation response. Taking together the data of all experimental conditions reported in Supplementary Table 1, there is a clear linear correlation between Chl and Car content for *Nannochloropsis* cells, which is maintained independently from all the differences in growth parameters (Figure 4). This can be explained by the fact that pigment binding proteins in the photosynthetic apparatus bind Chl and Car with specific ratios and when their content changes both pigments are equally affected. It is interesting to observe that the tendency line trends to 0.01 pg Car/cell for a 0 Chl content, suggesting the presence of a carotenoid pool not bound to proteins of the photosynthetic apparatus. This pool might be free in membranes and active in scavenging ROS (Dall’Osto et al., 2010; Dell Aglio, 2021). Microalgae, including *Nannochloropsis*, can accumulate carotenoids in oil bodies, but this occurs especially under prolonged stress, and this is likely not influential here since all cultures considered were under active growth conditions.

**FIGURE 5:**
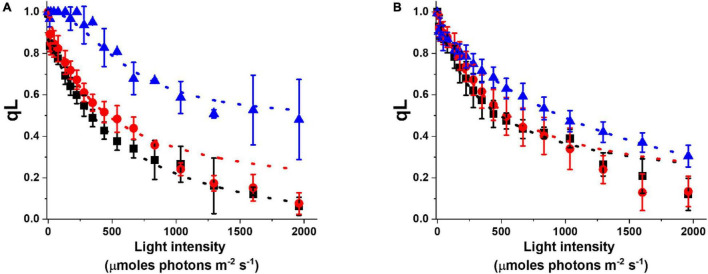
Photochemical activity of *Nannochloropsis* cells in different lab-scale conditions. Photochemical activity is expressed as the fluorescence parameter qL as a function of irradiance. Data were collected for cells grown in some of the conditions listed in Supplementary Table 1. Data refer to lab-scale cultures grown diluted (<25 × 10^6^ cells/ml) in flasks **(A)** and dense (>50 × 10^6^ cells/ml) in glass tubes with air bubbling **(B)**, and acclimated to 10 (black squares), 100 (red dots), and 1,000 (blue triangles) μmoles of photons m^–2^ s^–1^.

The acclimation response impacts not only pigment composition but also photosynthetic functionality. This can be evidenced by analyzing the fluorescence parameter qL, which enables to estimate the photochemical activity. qL is 1 when all reaction centers are open and available for photochemical reactions and it decreases when cells are exposed to increasing illumination, trending to 0 when the photosynthetic capacity is saturated (Baker, 2008). In cultures acclimated to more intense illumination, qL values are generally higher, suggesting that these cells have a higher photochemical capacity and thus a stronger illumination is needed to reach saturation (Figure 5A). This common trend is observed under different culture conditions, but while the difference is strong in flask cultures, the acclimation of photosynthetic capacity is smaller in cultures cultivated at higher density in glass tubes with air bubbling (i.e., Multicultivator from Photon System Instruments, PSI, Supplementary Table 1), suggesting they are perceiving on average less light than the incident irradiation (Figure 5B).

### Acclimation of Photosynthesis in Microalgae Cultivated Outdoors

Many of the parameters described above are affected when microalgae are cultivated outdoors, starting from the illumination intensity that changes continually during days, weeks, and seasons, but their impact on microalgae acclimation and photosynthetic functionality is still insufficiently investigated even if *in situ* probes for a continuous monitoring of photosynthetic efficiency were developed (Masojídek et al., 1999, 2004, 2010).

**FIGURE 6:**
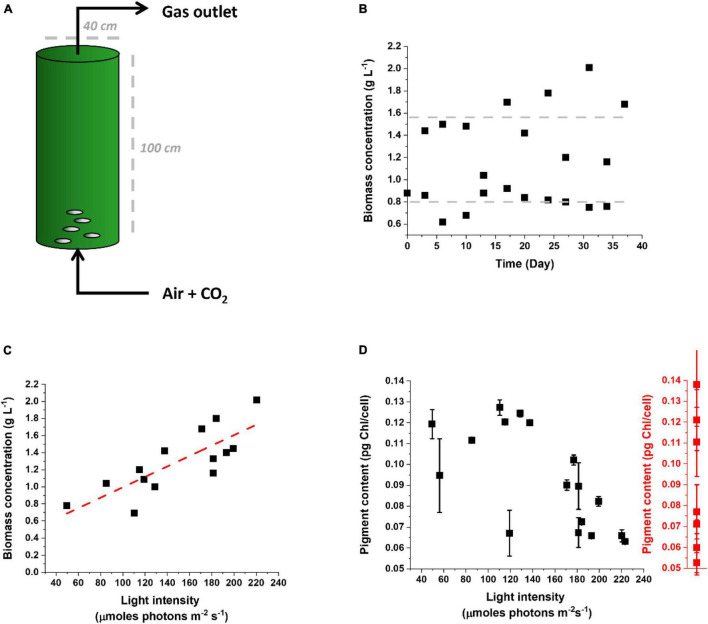
Cultivation of *Nannochloropsis gaditana* in a pilot PBR outdoor. **(A)** Scheme of the pilot PBR used to cultivate *Nannochloropsis* outdoor. Nutrients and CO_2_ were provided in excess to highlight the effect of the natural light supply rate on productivity. **(B)** The culture was operated in a semicontinuous mode for more than 1 month, restoring a biomass concentration of 0.8 g L^–1^ every other day. **(C)** Correlation between biomass concentration and light intensity reaching the culture. Light intensity was calculated as the average of illumination over 24 h. The linear trend is highlighted by a red dashed line. **(D)** Correlation between the pigment content of *Nannochloropsis* cells cultivated outdoor in the pilot PBR of panel **(A)** and the light intensity reaching the culture. The range of values of Chl content observed for the same cells cultivated in the lab and exposed to different irradiances is indicated by the red plot on the right. Data for the latter were collected for the lab conditions summarized in Supplementary Table 1.

It is instead highly interesting and stratetic comparing the response of microalgae cultivated outdoors to lab-scale cultures and assessing whether the response to outdoor cultivation corresponds to the phenomena observed indoors, on the path to drive improvements of photosynthetic productivity in the intended environment of cultivation at industrial scale. Figure 6 shows data from *Nannochloropsis gaditana* cultivated outdoors in a pilot PBR (Figure 6A) in semicontinuous mode to maintain high cellular concentration and biomass productivity (Figure 6B) while providing nutrients and CO_2_ in excess [Perin et al. (2015) as a reference on media composition]. These experiments were carried out in a greenhouse in February–April 2021 and while the temperature was controlled at 22°C, irradiance was natural.

Culture productivity was variable during different days, as expected, but it emerged a clear correlation with light intensity (Figure 6C). This suggests that, at least in the tested growth conditions, the culture was light-limited, and more available irradiation corresponded to higher productivity. Since productivity is limited from light availability, clearly any optimization of photon-to-biomass conversion efficiency would have a positive effect on yield.

The Chl content of cells in outdoor conditions showed large variability, going from 0.06 to 0.12 pg Chl/cell (Figure 6D). It is interesting to observe that this range of variability corresponds to the differences observed at the lab scale for cells exposed to different irradiances (Figures 3A, 6D). This suggests that microalgae cultivated outdoors activate an acclimation response as extensive as observed indoors and thus that this response is indeed largely activated when they are cultivated for an extended time at an industrial scale.

The pigment content measured outdoors also shows a correlation with the light intensity experienced by the culture, with higher average light intensities stimulating a decrease in Chl content. This is not true for external illumination below 100 μmoles of photons m^–2^ s^–1^, suggesting that below this value the maximal Chl content per cell was already reached. This is consistent with a similar conclusion obtained from lab-scale experiments, considering that, because of the culture density, the average illumination perceived by the cells is well below 10 μmoles of photons m^–2^ s^–1^ (Figure 3A).

Overall, these data highlight that acclimation is a phenomenon activated by microalgae also upon cultivation in PBR outdoors. In fact, while data at the lab scale are still significant to investigate the biological response in detail, parameters in outdoor conditions have other kinetics (e.g., light variability), whose impact should be investigated more thoughtfully. A better understanding of these responses in outdoors environments is strategic to drive microalgae genetic modifications effective in industrial conditions.

## Conclusion

Photon-to-biomass conversion efficiency is seminal for microalgae biomass productivity, given large-scale cultures are often light-limited, as also demonstrated here with data from an outdoor pilot PBR. Mechanisms for the regulation of photosynthesis, by modulating the ability of microalgae to convert light energy into biomass or dissipate the irradiance in excess as heat, have a fundamental impact on cell fitness and consequently productivity.

The impact of both short- and long-term regulatory mechanisms of photosynthesis during industrial cultivation is still under-considered and the biological response of microalgae at industrial-scale is still largely obscure. Further efforts are thus needed to clarify also the impact of an inhomogeneous distribution of carbon and nutrients on photosynthesis acclimation at the industrial scale and also to fully understand the impact of regulatory mechanisms investigated at the lab scale.

As domesticated crops must show improved performances on the field, also optimized microalgae strains must have improved biomass productivity in the intended cultivation environment of PBRs/ponds. Despite biotechnology to domesticate microalgae photosynthesis has been largely deployed for over one decade, tested modifications are still at the stage of a proof-of-concept at the lab scale. One of the reasons is the high costs for building and operating pilot-scale microalgae cultivation facilities, preventing the collection of data at a more relevant scale of cultivation. The sooner this further layer of complexity will be considered, the faster biotechnology will be able to deploy more effective strategies of metabolic engineering of photosynthesis to ultimately close the gap with the theoretical potential of microalgae-biomass-based technologies.

## Data Availability Statement

Publicly available datasets were analyzed in this study. Data are found in original publications cited.

## Author Contributions

GP and TM wrote the manuscript. All authors analyzed the data presented and approved the submitted version.

## Conflict of Interest

The authors declare that the research was conducted in the absence of any commercial or financial relationships that could be construed as a potential conflict of interest.

## Publisher’s Note

All claims expressed in this article are solely those of the authors and do not necessarily represent those of their affiliated organizations, or those of the publisher, the editors and the reviewers. Any product that may be evaluated in this article, or claim that may be made by its manufacturer, is not guaranteed or endorsed by the publisher.
