# Supplementary material for: Knowledge of Regulation of Photosynthesis in Outdoor Microalgae Cultures Is Essential for the Optimization of Biomass Productivity
Source: Front Plant Sci. 2022 Apr 4;13:846496. doi: 10.3389/fpls.2022.846496 (PMC9014180; doi:10.3389/fpls.2022.846496)
Supplement: Supplementary file 1 [file Data_Sheet_1.pdf]

Supplementary material

REGULATION OF PHOTOSYNTHESIS IN OUTDOOR MICROALGAE CULTURES IS SEMINAL FOR BIOMASS PRODUCTIVITY

Giorgio Perin[1], Francesca Gambaro[1] and Tomas Morosinotto[1,*]

1. Department of Biology, University of Padova, Padova, Italy

* corresponding author, e-mail: tomas.morosinotto@unipd.it

Supplementary tables

Supplementary table S1. List of growth conditions in which data for figures 3, 4 and 5 were collected. Flask, limiting carbon source. Orbital agitation provides mixing; Multicultivator, from Photon System Instruments (PSI), 2-cm diameter glass tubes with carbon source > flask. Mixing is provided by air bubbling; Lab-scale PBR, 5-cm diameter Drechsel bottle where mixing is provided by air bubbling + 5% CO_2. Media: F/2 contains 32 g/L sea salts (Sigma Aldrich), 40 mM Tris-HCl (pH 8) and Guillard's (F/2) marine water enrichment solution (Sigma Aldrich). F/2 enriched was supplemented with added nitrogen, phosphate and iron sources (0.75, 0.05 and 0.0063 g L^{-1} final concentration, respectively for $NaNO_3$, NaH_2PO_4 and $FeCl_3 \cdot 6H_2O$). Nitrogen (N) and phosphorous (P) limitation was achieved switching from a 8.82 to 2.65 mM and from 0.42 to 0.1 mM concentration, respectively for $NaNO_3$, NaH_2PO_4.

ID growth condition (#)	Light intensity (µmol photons m^{-2} s^{-1})	Cultivation system	CO_2 supply	Medium	Cultivation mode	Cells concentration (10^6 cells/ml)	Reference
**1**	10	Flask	Air duffusion	F/2	batch	10	(Meneghesso et al., 2016)
**2**	100	Flask	Air duffusion	F/2	batch	10	(Meneghesso et al., 2016)
**3**	1000	Flask	Air duffusion	F/2	batch	10	(Meneghesso et al., 2016)
**4**	10	Multicultivator (PSI)	Air bubbling	F/2	batch	10	(Alboresi et al., 2016)
**5**	100	Multicultivator (PSI)	Air bubbling	F/2	batch	10	(Alboresi et al., 2016)
**6**	1000	Multicultivator (PSI)	Air bubbling	F/2	batch	10	(Alboresi et al., 2016)
**7**	10	Multicultivator (PSI)	Air bubbling	F/2 enriched	batch	10	(Meneghesso et al., 2016)
**8**	100	Multicultivator (PSI)	Air bubbling	F/2 enriched	batch	10	(Meneghesso et al., 2016)
**9**	1000	Multicultivator (PSI)	Air bubbling	F/2 enriched	batch	10	(Meneghesso et al., 2016)
**10**	400	Lab-scale PBR	Air bubbling + 5% CO_2	F/2 enriched	semi-continuous	250	(Perin et al., 2017)
**11**	400	Lab-scale PBR	Air bubbling + 5% CO_2	F/2 enriched	semi-continuous	150	(Perin et al., 2017)
**12**	1200	Lab-scale PBR	Air bubbling + 5% CO_2	F/2 enriched	semi-continuous	250	(Perin et al., 2017)
**13**	400	Lab-scale PBR	Air bubbling + 5% CO_2	F/2 in N limitation	semi-continuous	150	(Fattore et al., 2021)
**14**	400	Lab-scale PBR	Air bubbling + 5% CO_2	F/2 in P limitation	semi-continuous	150	(Fattore et al., 2021)

References

Alboresi, A., Perin, G., Vitulo, N., Diretto, G., Block, M., Jouhet, J., et al. (2016). Light Remodels Lipid Biosynthesis in Nannochloropsis gaditana by Modulating Carbon Partitioning between Organelles. *Plant physiology* 171, 2468–82. doi:10.1104/pp.16.00599.

Fattore, N., Bellan, A., Pedroletti, L., Vitulo, N., and Morosinotto, T. (2021). Acclimation of photosynthesis and lipids biosynthesis to prolonged nitrogen and phosphorus limitation in Nannochloropsis gaditana. *Algal Research* 58, 102368. doi:10.1016/J.ALGAL.2021.102368.

Meneghesso, A., Simionato, D., Gerotto, C., la Rocca, N., Finazzi, G., and Morosinotto, T. (2016). Photoacclimation of photosynthesis in the Eustigmatophycean Nannochloropsis gaditana. *Photosynthesis Research* 129, 291–305. doi:10.1007/s11120-016-0297-z.

Perin, G., Simionato, D., Bellan, A., Carone, M., Occhipinti, A., Maffei, M. E., et al. (2017). Cultivation in industrially relevant conditions has a strong influence on biological properties and performances of Nannochloropsis gaditana genetically modified strains. *Algal Research* 28, 88–99. doi:10.1016/j.algal.2017.10.013.
